# ESPO and Biological Sciences Section Symposium: Highlighting the Future Leaders of Geroscience

**DOI:** 10.1093/geroni/igad104.1812

**Published:** 2023-12-21

**Authors:** Matt Yousefzadeh, Bhaswati Ghosh

## Abstract

The Emerging Scholars Symposium (ESPO) is organized by post-docs and PhD students from GSA’s BS ESPO Committee. The goal of this symposium is to foster the next generation of aging researchers by recruiting trainees and early career investigators as speakers from contributed abstracts.

